# A case report of untreatable HIV infection in Harare, Zimbabwe

**DOI:** 10.4102/sajhivmed.v20i1.885

**Published:** 2019-06-27

**Authors:** Cleophas Chimbetete, Linda Chirimuta, Margaret Pascoe, Olivia Keiser

**Affiliations:** 1 Institute of Global Health, University of Geneva, Geneva, Switzerland; 2 Newlands Clinic, Harare, Zimbabwe

**Keywords:** Dolutegravir, Resistance, Untreatable HIV, Zimbabwe, ART programmes

## Abstract

**Introduction:**

Zimbabwe, like other resource limited countries, manages HIV infection using the public health approach with standard antiretroviral therapy (ART) regimens for first, second and third-line treatment. Third-line ART is the last available treatment option and is based on dolutegravir and darunavir use after HIV drug resistance testing.

**Patient Presentation:**

We report here a 17-year-old patient on dolutegravir (DTG) and Darunavir based third-line antiretroviral therapy (ART) previously exposed to raltegravir who develops multidrug resistance HIV to the four ART classes available in Zimbabwe.

**Management and Outcome:**

A trophism assay revealed that patient has CXCR4 trophic virus and hence will not benefit from Maraviroc. Patient is currently stable and receiving a holding regimen of abacavir, lamivudine and lamivudine.

**Conclusion:**

This is the first documented case of multiclass resistance to the four available ART classes in Zimbabwe. The development and transmission of multiclass HIV drug resistance in resource limited settings has potential to undo the gains of national ART programs. There is need to ensure optimum adherence to ART even in the era of DTG.

## Background

Widespread availability of antiretroviral therapy (ART) has transformed a positive HIV diagnosis from being a death sentence into a chronic manageable disease. To date, no cure exists for HIV, and hence patients must remain on effective ART for the rest of their lives, that makes the development of drug resistance a major public health concern. Sustained viral suppression is of paramount importance if drug resistance is to be prevented. Strategies to ensure optimal adherence to ART are, therefore, an important component of HIV care and treatment. Antiretroviral therapy resistance limits further treatment options, increases treatment programme costs and drug resistance may even be transmitted to others.^1^ The rising prevalence of HIV drug resistance poses a great threat to the HIV response and has the potential to drive increase in mortality and HIV incidence.^2^ Several risk factors for the development of HIV drug resistance among patients on ART have been identified.^3^

HIV treatment in Zimbabwe is based on a public health approach using standard national treatment guidelines.^4^ Treatment guidelines have periodically changed and are guided by the World Health Organization (WHO). In 2015, Zimbabwe introduced third-line ART in the national programme. Patients failing second-line ART are referred for specialist assessment that includes viral load (VL) and genotype testing prior to recommending third-line medicines. Adherence needs to be reinforced at all times.^4^

We report the first case of documented four-class HIV drug resistance in Zimbabwe that highlights the possibility of third-line ART failure and transmission of untreatable HIV in resource-limited settings.

## Case report

We report the case of an adolescent girl born in July 2000. She tested positive for HIV infection in 2009 and was enrolled into care at Newlands Clinic on 30 July 2009. She is the last born in a family of three children, a paternal orphan and stays with her mother. She was vertically infected, and her mother is accessing ART at the same treatment centre. Both her siblings are HIV negative. She commenced first-line ART on 28 August 2009. Table 1 summarises ART regimens received over time and the reasons for regimen changes.

**TABLE 1:** Antiretroviral therapy history by regimen.

ART regimen	Start date	End date	Reason for switch
d4T/3TC/NVP	28/08/2009	30/07/2010	Guideline change
AZT/3TC/NVP	30/07/2010	10/04/2012	Treatment failure
LPV/r/AZT/3TC	10/04/2012	20/01/2015	Guideline change
ATV/r/3TC/ABC	20/01/2015	12/08/2015	Treatment failure
RAL/DRV/r/3TC	12/08/2015	28/07/2016	Clinic decision
DTG/DRV/r/3TC	28/07/2016	21/03/2017	Poor adherence
3TC Monotherapy	21/03/2017	23/01/2018	Change to effective regimen
DTG/DRV/r/3TC	23/01/2018	12/11/2018	Changed to holding regimen
ABC/3TC/AZT	12/11/2018	Current	-

ART, antiretroviral therapy; d4T, stavudine; AZT, zidovudine; 3TC, lamivudine; NVP, nevirapine; ABC, abacavir; r, ritonavir; DRV, darunavir; ATV, atazanavir; LPV, lopinavir; RAL, raltegravir.

**FIGURE 1:**
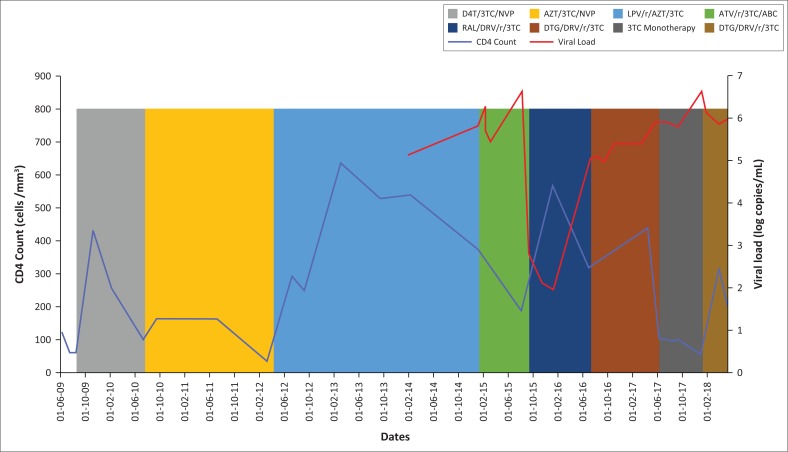
CD4 count, viral load and antiretroviral therapy regimens over time.

Monitoring for ART treatment success was done clinically and immunologically since the initiation of treatment. Routine VL monitoring was added in January 2014. Figure 1 highlights the patient’s CD4, VL and ART regimens over time.

### HIV drug resistance testing and third-line response

A genotypic resistance test was performed on 31 March 2015 after second-line ART failure. Results of the test were interpreted using the Stanford HIV drug resistance guide. We found four major protease inhibitor (PI) resistance-associated mutations (RAMs), that is, M46I, I54V, L76V and V82A. The PI RAMs conferred high-level resistance to atazanavir (ATV), lopinavir, indinavir and saquinavir.

There were three nucleoside reverse transcriptase (NRTI) RAMs, that is, M41L, M184V and T215F, and three non-NRTI RAMs, that is, A98G, K103N and E138A. The RAMs conferred intermediate resistance to abacavir, zidovudine, stavudine, didanosine and rilpivirine. There was high-level resistance to emtricitabine, lamivudine, efavirenz and nevirapine. The virus had low-level resistance to tenofovir and etravirine. Table 2 summarises results of the resistance tests conducted during the course of patient management.

**TABLE 2:** HIV drug resistance test results.

2015			2016	
Medicines	Mutations	Description of resistance	Mutations	Description of resistance
**NRTI**	*M41L, M184V, T215F*		*M41L, M184V, T215F*	
Zidovudine		Intermediate		Intermediate
Lamivudine		High level		High level
Abacavir		Intermediate		Intermediate
Emtricitabine		High level		High level
Tenofovir		Low level		Susceptible
**NNRTI**	*A98G, K103N, E138A*		*A98G, K103N, E138A*	
Nevirapine		High level		High level
Efavirenz		High level		High level
Rilpivirine		Intermediate		Intermediate
Etravirine		Low level		Low level
**PI**	*M46I, I54V, V82A*		*M46I, I54V, V82A, L76V*	
Lopinavir		High level		High level
Atazanavir		High level		High level
Darunavir		Susceptible		Intermediate
**INSTI†**			*E138K, G140A, Q148R*	
Elvitegravir				High-level resistance
Raltegravir				High-level resistance
Darunavir				High-level resistance

NRTI, nucleoside reverse transcriptase inhibitors; NNRTI, non-nucleoside reverse transcriptase inhibitors; INSTI, integrase strand transfer inhibitor.

† , Test done on 14 June 2018.

She was started on third-line ART in August 2015. She has had challenges with treatment adherence because of the high pill burden, and received 3TC monotherapy as a holding therapy from March 2017 (VL was 255 397 copies/mL) to January 2018. She was treated for pulmonary tuberculosis (TB) from 08 August 2017 to 23 January 2018. The TB diagnosis was made based on loss of weight and suggestive chest X-ray findings. She improved clinically on TB treatment, and after completing 6 months of therapy, she recommenced third-line therapy with ritonavir-boosted darunavir, lamivudine and dolutegravir (DTG). She came daily to the clinic for a nurse to observe her and to take third-line medicines for 16 weeks, but her VL remained very high.

An integrase strand transfer inhibitor (INSTI) resistance test was then performed on 14 June 2018. Results showed three integrase inhibitor major RAMs, that is, E138K, G140A and Q148R. The RAMs conferred high-level resistance to DTG, raltegravir (RAL) and elvitegravir (ELV). Trophism assay was performed, and results showed that unfortunately the patient is CXCR4 trophic and hence maraviroc is unlikely to work. The recently approved post-attachment inhibitor, ibalizumab, is not available in the country. She was commenced on a holding regimen of ABC, 3TC and AZT, and her latest VL done on 12 November 2018 was 771 334 copies/mL. Her mother is virologically suppressed on a second-line ART regimen of ATV or ritonavir, AZT and 3TC.

## Ethical consideration

Analysis of routine clinical data is approved by the Medical Research Council of Zimbabwe as part of a larger study, International Epidemiological Databases to Evaluate AIDs (IeDEA Collaboration) (approval no. MRCZ/A/1336). Verbal assent from adolescent and written informed consent from parent were obtained.

## Discussion

To our knowledge, this is the first report of a patient with a virus that has developed multi-class drug resistance to all four standard classes of ART, including INSTIs, in Zimbabwe. This patient has HIV with high-level resistance to DTG after previous exposure to RAL. Recently, a case of multi-drug resistant HIV, including resistance to INSTIs, was reported from Botswana^5^ and a similar case was reported earlier in South Africa.^6^ Multi-drug resistant HIV could have developed because of a variety of factors, including poor adherence to ART and inadequate psychosocial support – issues which are frequently encountered among adolescents living with HIV.^7^ In this case, poor adherence was mainly because of poor family support and lack of motivation for ART when the patient felt clinically well. Poor adherence to previous ART regimens could have led to exposure to DTG monotherapy. Previous studies have shown that monotherapy with DTG has a high rate of resistance selection in the integrase gene through different pathways in case of virologic failure.^8^

Integrase strand transfer inhibitors are one of the newest class of antiretroviral drugs to be approved for HIV treatment and act by inhibiting the essential HIV protein integrase from inserting the viral DNA genome into the host cell’s chromatin. Raltegravir and EVG have been successful in clinical settings, but have low genetic barriers to resistance. Dolutegravir is known to have a very high genetic barrier to resistance and retains activity against RAL- and EVG-resistant viruses.^9,10^ Zimbabwe has not yet adopted the use of DTG as part of the preferred first-line ART regimens.

## Conclusion

This is the first case of recorded four-class HIV drug resistance in Zimbabwe. This adolescent girl cannot be effectively treated with any of the currently available ART regimens in Zimbabwe. Prevention measures such as family planning intervention and safe sex counselling are being taken to minimise the risk of transmission of this multi-class resistant virus.

This case emphasises the need for health workers to continue providing adherence counselling and support for patients who are on ART. Transmission of four-class-resistant HIV is a potential public health disaster.
